# Gene expression is associated with virulence in murine macrophages infected with *Leptospira* spp

**DOI:** 10.1371/journal.pone.0225272

**Published:** 2019-12-04

**Authors:** Erivelto Corrêa de Araújo Junior, Leandro Encarnação Garcia, Matheus Janeck Araújo, Itamar Souza Oliveira-Junior, Daniel Robert Arnold, Flavia Lombardi Lopes, Márcia Marinho

**Affiliations:** 1 Department of Production and Animal Health, São Paulo State University (Unesp), School of Veterinary Medicine, Araçatuba, SP, Brazil; 2 Department of Surgery, Discipline of Anesthesia, Pain and Intensive Medicine, Federal University of São Paulo, São Paulo, Brazil; Cornell University, UNITED STATES

## Abstract

*Leptospira* genus contains species that affect human health with varying degrees of pathogenicity. In this context, we aimed to evaluate the differences in the modulation of host gene expression by strains of *Leptospira* varying in virulence. Our data showed a high number of differentially expressed transcripts in murine macrophages following 6h of infection. *Leptospira* infection modulated a set of genes independently of their degree of virulence. However, pathway analysis indicated that Apoptosis, ATM Signaling, and Cell Cycle: G2/M DNA Damage Checkpoint Regulation were exclusively regulated following infection with the virulent strain. Taken together, results demonstrated that species and virulence play a role during host response to *Leptospira* spp in murine macrophages, which could contribute to understanding the pathogenesis of leptospirosis.

## Introduction

Leptospirosis disease can occur in different epidemiological conditions [1]. The genus *Leptospira* encompasses pathogenic and saprophytic species that differ in their ability to survive and colonize different environments and hosts [2]. *Leptospira* species are classified into three groups according to their pathogenic potential: virulent pathogenic, intermediate, and saprophytes [3]. Leptospirosis occurs mainly in vulnerable populations, including urban and rural dwellers [4] of tropical and subtropical developing countries [5–7]. It is a major public health problem, with a recent estimate of 1 million cases per year, and a mortality rate of 5 to 10% [4,8–9].

Leptospires are capable of infecting humans and many domestic and wild animals, survive and thrive in host tissues, escaping from the host's natural defense mechanisms. Transmission is based on direct or indirect contact with the urine of carriers (mainly rodents); the disease varied from sub-clinical to most serious cases, progressing to renal failure and pulmonary hemorrhage [1–10]. Rodents are natural reservoir for leptospires, in this work we use murine macrophage cell line to understand host-specific immune response against infection.

Host-specific immune response against pathogenic leptospires is poorly understood, particularly regarding susceptibility resistance to infection. For decades, adaptive humoral immunity was considered as the sole player in leptospirosis, but recent work points to a role for innate and adaptive immunity [11–14].

Phagocytosis is a mechanism to eliminate invading microbial pathogens at the early stages of infection in individuals without acquired immunity against the infecting agent, but pathogenic *Leptospira* can escape from complement attack and phagocytosis after infection [15–17]. Pathogenic *Leptospira* is also able to survive and replicate in human macrophages, but it is killed in murine macrophages [18]. LPSs of pathogenic *Leptospira* activate human macrophages only through the Toll-like receptor 2 (TLR2) while murine macrophages are activated through TLR2 and TLR4 [13–19]. Vernon Pauillac and Merian [20] have shown that mononuclear macrophages of peripheral blood of hamster infected with a virulent variant of *Leptospira interrogans* secrete proinflammatory cytokines (TNF-α) with a Th1 (IL-12) profile in the first hour, predominating until the fourth day after infection, whereas a Th2 profile appears after 24 hours of infection. In the early course of infection, leptospires survive and spread in the bloodstream before causing damage to target organs [21].

In this study, we applied microarray technology to comparatively analyze early changes in murine macrophages genes expression in response to *Leptospira* spp. with varied virulence, and to identify signaling pathways that play a role in an *in vitro* model of macrophageal infection.

## Results

### Data deposition

Microarray raw data files are available in Gene Expression Omnibus (GEO) and are accessible through GEO series number GSE105141 [22].

### Gene expression profile via microarray analysis

**Fig 1:**
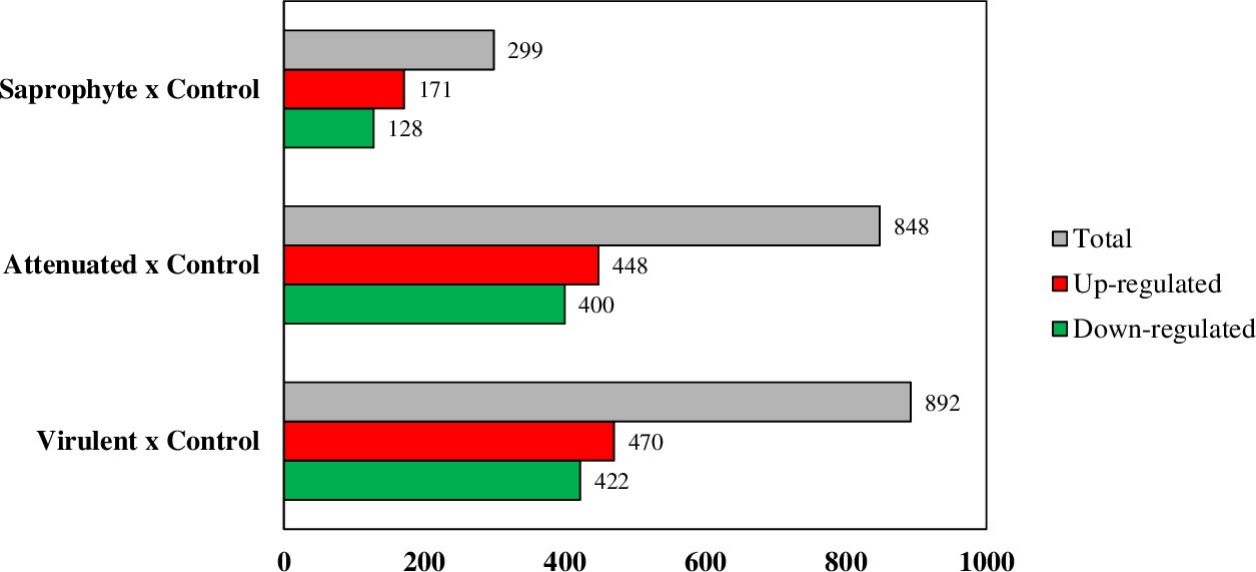
Differentially expressed genes after 6 hours of infection in murine macrophages J774A.1 with saprophytic, attenuated and virulent strains of *Leptospira* spp. The colored bars show the up-regulated (red) or down-regulated (green) genes and grey a total of genes. (n = 3 / assay, FDR-adjusted p<0.05, fold change ± 2).

Our data analysis found 892 genes in cells infected with saprophyte, attenuated and virulent leptospirosis compared to control. According to Fig 1, pathogenic leptospires modulates 892 genes (422 up and 470 down-regulated), attenuated leptospires modulates 848 genes (400 upregulated and 448 downregulated) and saprophyte 299 genes (128 upregulated and 171 downregulated) in a filter criterion of fold change ±2 and false discovery rate (FDR)<0.05 (Fig 1).

**Fig 2:**
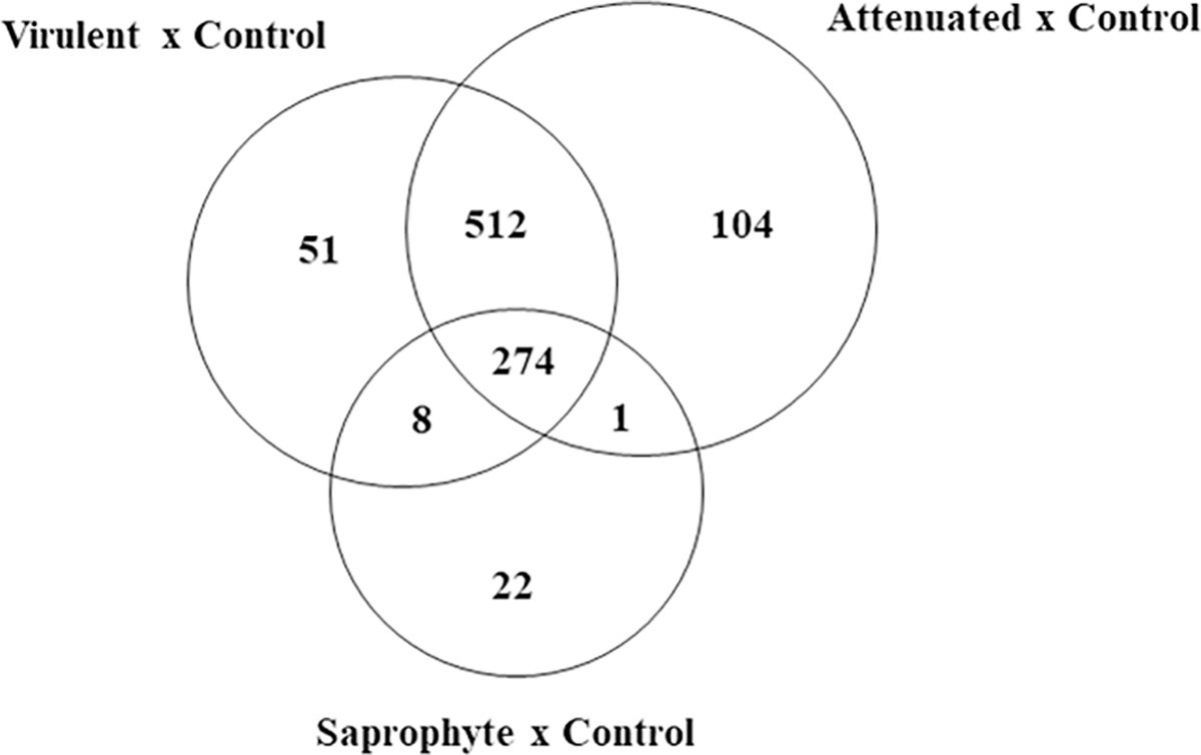
Venn diagram for differentially expressed genes after 6 hours of infection in murine macrophages J774A.1 with saprophytic, attenuated and virulent strains of *Leptospira* spp. Total number of canonical pathways (n = 3/treatment; FDR<0.05, fold change ± 2) in the contrasts Infected (Saprophyte; Attenuated and Virulent) vs. Non-infected Control.

Through treatment comparison by Venn diagram, we identify common and specific genes (Fig 2).

**Fig 3:**
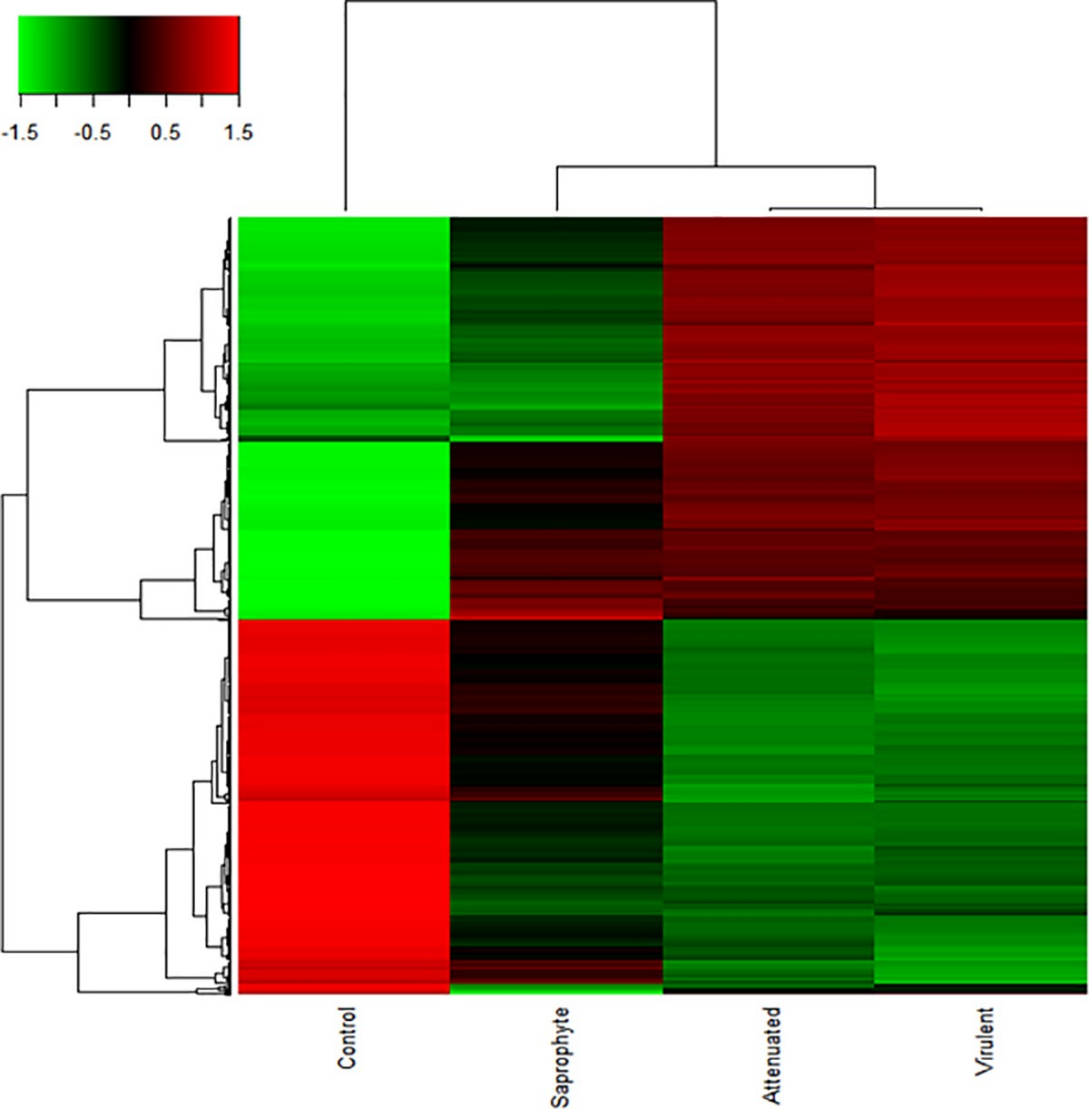
Heatmap of differentially expressed genes shows the average signal by macrophages at 6 hours of infection in different strains of *Leptospira* spp. The red color indicate increased expression, green color indicates the decreased expression as compared to control (n = 3/treatment; p-value<0.01; FDR<0.05; linear fold change ± 2).

A total of 274 genes were common to all infected cells, despite of strains, when compared to control. Virulent and culture-attenuated infected groups groups shared 512 genes in common, while eight genes were shared between virulent and saprophyte groups and only one gene between attenuated and saprophyte infected cells (S1 Table). Average signal (log2) of samples were hierarchically clustered using Pearson correlation and complete-linkage and it was observed again a clustering of samples based on species and virulence, with the virulent and culture-attenuated strains clustering closer together, followed bt the saprophyte strain (Fig 3).

In Table 1 we depict the top 9 DEGs in response to infection. These genes are present in several pathways and biological processes involved in acute inflammatory response.

**Table 1:** Top modulated transcripts in murine macrophages following 6h of infection with saprophyte, culture-attenuated and virulent strains of *Leptospira* spp.

Regulation	Gene Symbol	FDR-adjusted p-value	FC (Sap vs. CT)	FC (Att vs. CT)	FC (Vir vs. CT)
**Up**	Il1b	0,000037	202,85	253,12	259,03
	Il1a	0,000004	42,58	120	127,28
	Saa3	0,000003	58,32	113,49	95,44
	Il6	0,000005	17,92	90,05	93,09
	Ccl5	0,000008	15,72	56,83	56,56
	Ptgs2	0,000011	38,21	52,72	56,33
	Nos2	0,000008	6,83	48,29	51,82
	Cxcl10	0,000035	8	45,54	51,25
	Ifit1	0,000217	6,16	33,6	37,61
**Down**	Rasgrp3	0,000059	-6,97	-9,16	-7,8
	Ighm	0,000045	-4,88	-8,22	-7,91
	Hal	0,000022	-4,52	-6,78	-7,01
	Cxcr4	0,000133	-5,65	-5,93	-6,11
	Klhl24	0,000053	-4,81	-5,77	-5,76
	Il18rap	0,000074	-3,7	-5,55	-5,31
	Nrcam	0,000032	-3,37	-5,43	-5,05
	Il1rl1	0,000028	-3,24	-5,35	-4,53
	Ankrd44	0,000476	-2,68	-5,3	-4,67

(FC = fold change; SAP = saprophyte; Att = attenuated; Vir = virulent)

### Analysis of signaling pathways

For functional enrichment of the differentially expressed genes obtained for each treatment, the Ingenuity Pathway Analysis (IPA) software was employed, Core Analysis was performed to identify relevant biological pathways to all 3 strains using the -log BH p-value > 1.3 (equivalent to a p-value <0.01).

### Specific pathways modulated by the virulent strain

**Fig 4:**
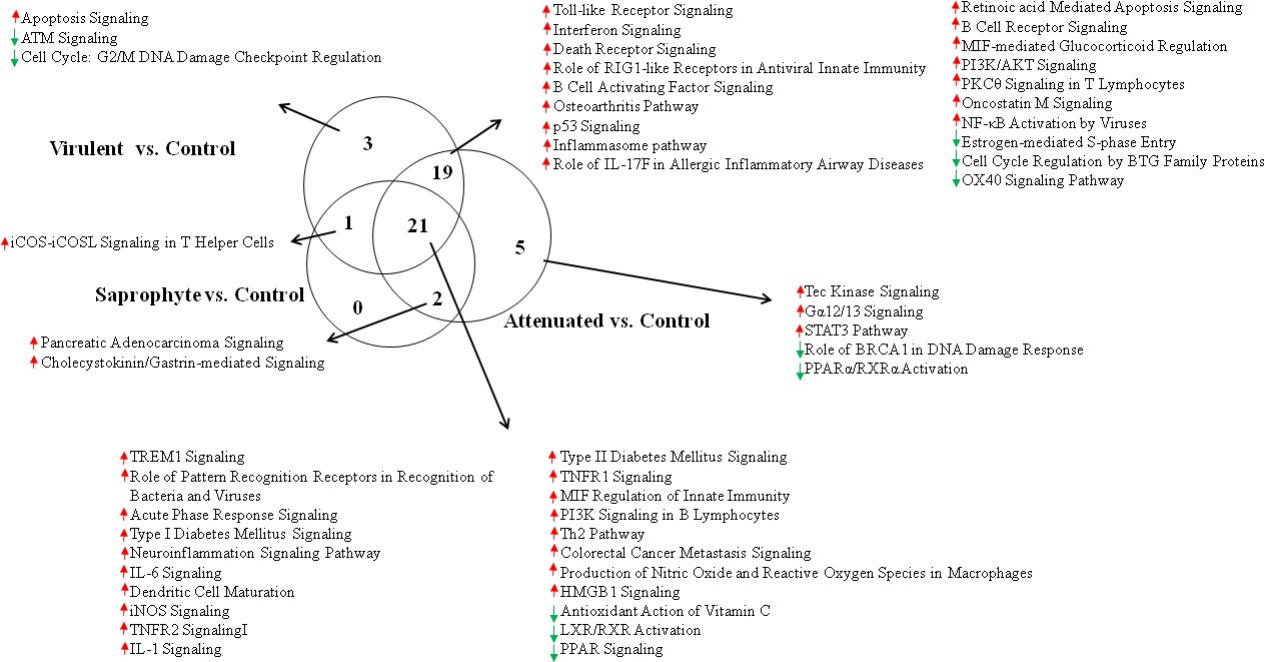
Venn diagram for pathways of modulated macrophages at 6 hours of infection with different strains of Leptospira spp. Total number of canonical pathways (n = 3/treatment; FDR<0.05, fold change ± 2) in the contrasts Infected (Saprophyte; Attenuated and Virulent) vs. Non-infected Control.

Several pathways were identified as regulated by the virulent strain, however the Apoptosis pathway, ATM signaling and Cell Cycle: G2 / M DNA Damage Checkpoint Regulation, were exclusively expressed and affected by treatment with the virulent strain (Fig 4 and S1 Fig).

**Fig 5:**
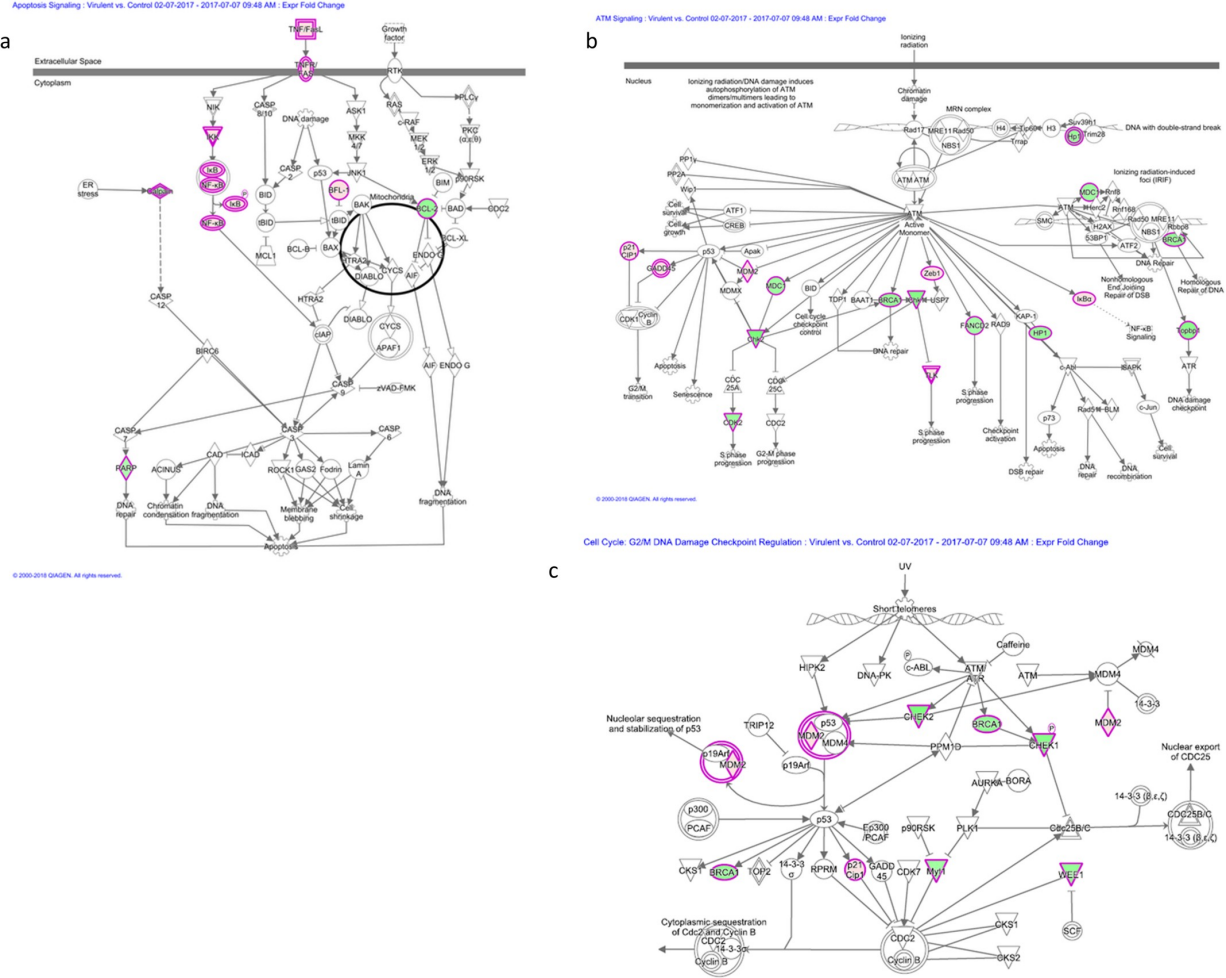
Canonical signaling pathway obtained by the IPA (Ingenuity Pathway Analysis software. Red and green indicate upregulated and down-regulated genes, respectively, compared to control group, and belongs to datasets of DEGs virulent vs. control assays. Color intensity corresponds to the degree of up or downregulation (fold-change). White represents the known genes of the pathway without identification in the transcriptomic analysis. **Panel A)** Canonical signaling pathway of Apoptosis of *in vitro* macrophages. **Panel B)** Canonical signaling of ATM of *in vitro* macrophages. **Panel C)** Canonical signaling pathway of Cell Cycle: G2 / M DNA Damage Checkpoint Regulation of *in vitro* macrophages.

In the apoptosis pathway, the major up-regulated genes were FAS, IKBKE, NFKB1, NFKBIA, NFKBIB, NFKNID, NFKBIE, TNF, TNFRSF1B; downregulated transcripts were BCL2, CAPN2 and PARP1 (Fig 5A). In the ATM signaling pathway, the upregulated transcript genes were CDKN1A, GADD45G, MDM2, NFKBIA and TLK2; downregulated transcripts were BRCA1, CBX5, CDK2, CHEK1, CHEK2, FANCD2, MDC1 and TOPBP1 (Fig 5B).

The upregulated genes of the Cell Cycle: G2 / M DNA Damage Checkpoint Regulation pathway were CDKN1A and MDM2; downregulated transcripts were BRCA1, CHEK1, CHEK2, PKMYT1 and WEE1 (Fig 5C).

### Validation of microarray data by qRT-PCR

**Fig 6:**
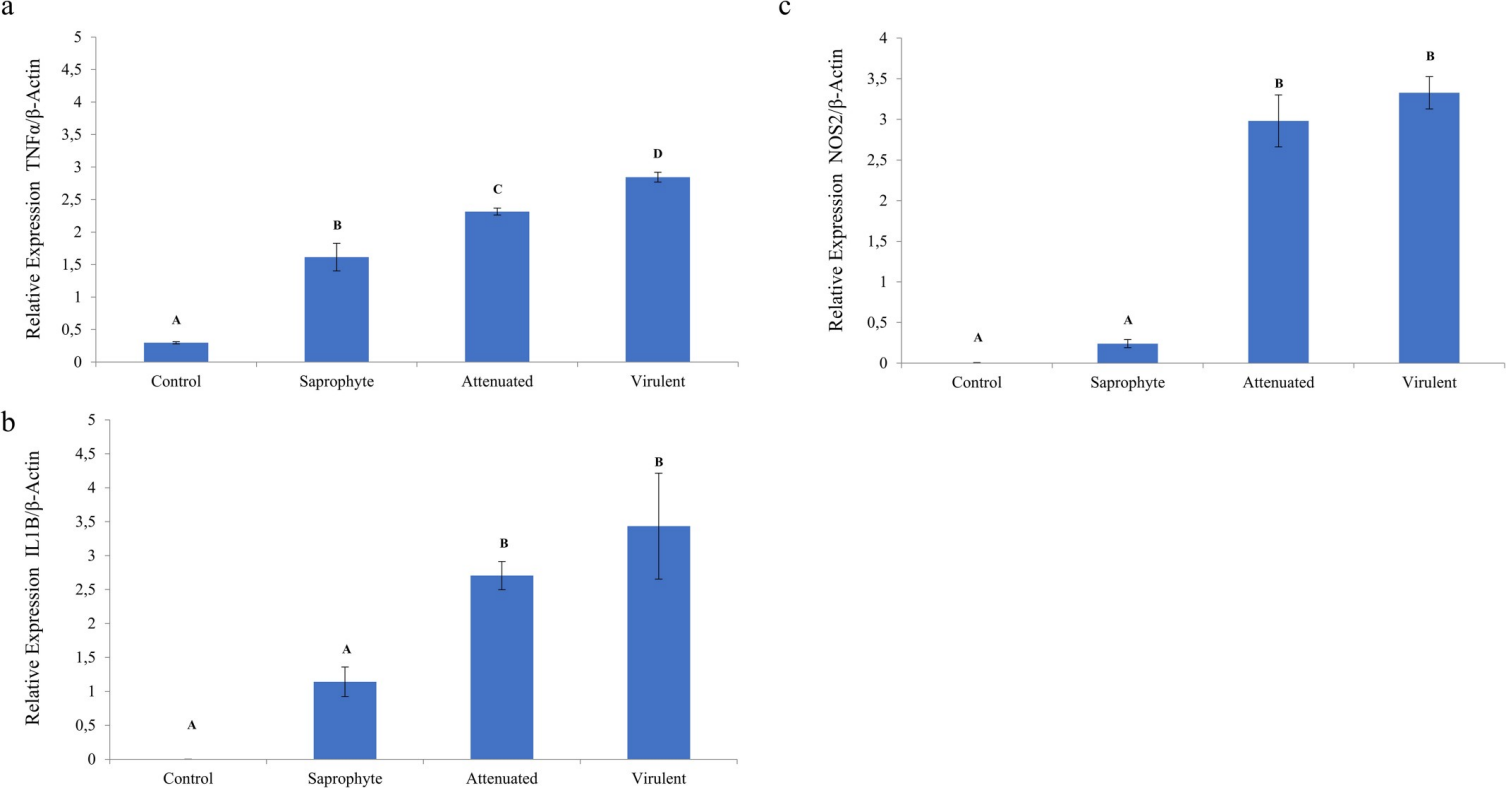
qRT-PCR of mRNA expression levels in infected macrophages with different strains of Leptospira compared to non-infected controls. Panel A) Relative expression of TNF-α in saprophyte, culture-attenuated and virulent compared to control. Panel B) Relative expression of IL-1β in saprophyte, culture-attenuated and virulent compared to control. Panel C) Relative expression of NOS2 in saprophyte, culture-attenuated and virulent compared to control (p <0.05). Different superscript letters differs significantly (p <0.05).

Infection with 10^7^ of virulent and attenuated (*L*. *interrogans* serovar Copenhageni) and saprophytic (*L*. *biflexa* serovar Patoc), induced significant increase of TNF-α expression in murine macrophages (p <0.0001) compared to control. Regarding expression of IL-1β and NOS2, a similar expression profile was observed between Control and Saprophy, which differed from the profile found in the Attenuated and Virulent samples. The comparative analysis of the expressed values for IL-1β and NOS2 were statistically different between the assays, compared to the attenuated and virulent strains, differing when compared to the control groups and infection with the saprophytic strain. Differently from the observed TNF expression results, there was significant difference across all assays (Fig 6A–6C).

## Discussion

In this study, we took an *in vitro* approach to analyze the trancriptomic profiles of macrophages in response to saprophytic, culture-attenuated and virulent samples of *Leptospira* spp, to gain a better understanding of the disease’s molecular mechanisms and pathways.

TREM-1 signaling was the most significant pathway modulated by all three strains. TREMs are a family of recently discovered receptors of the immunoglobulin superfamily, expressed on various cells of the myeloid lineage, which play important roles in innate immune responses, such as activating inflammatory responses and contributing to septic shock response in microbial-mediated infections [23–24]. Targeted activation of TREM-1 in our study appears to be a first line inflammatory response to the genus, regardless of virulence.

**Fig 7:**
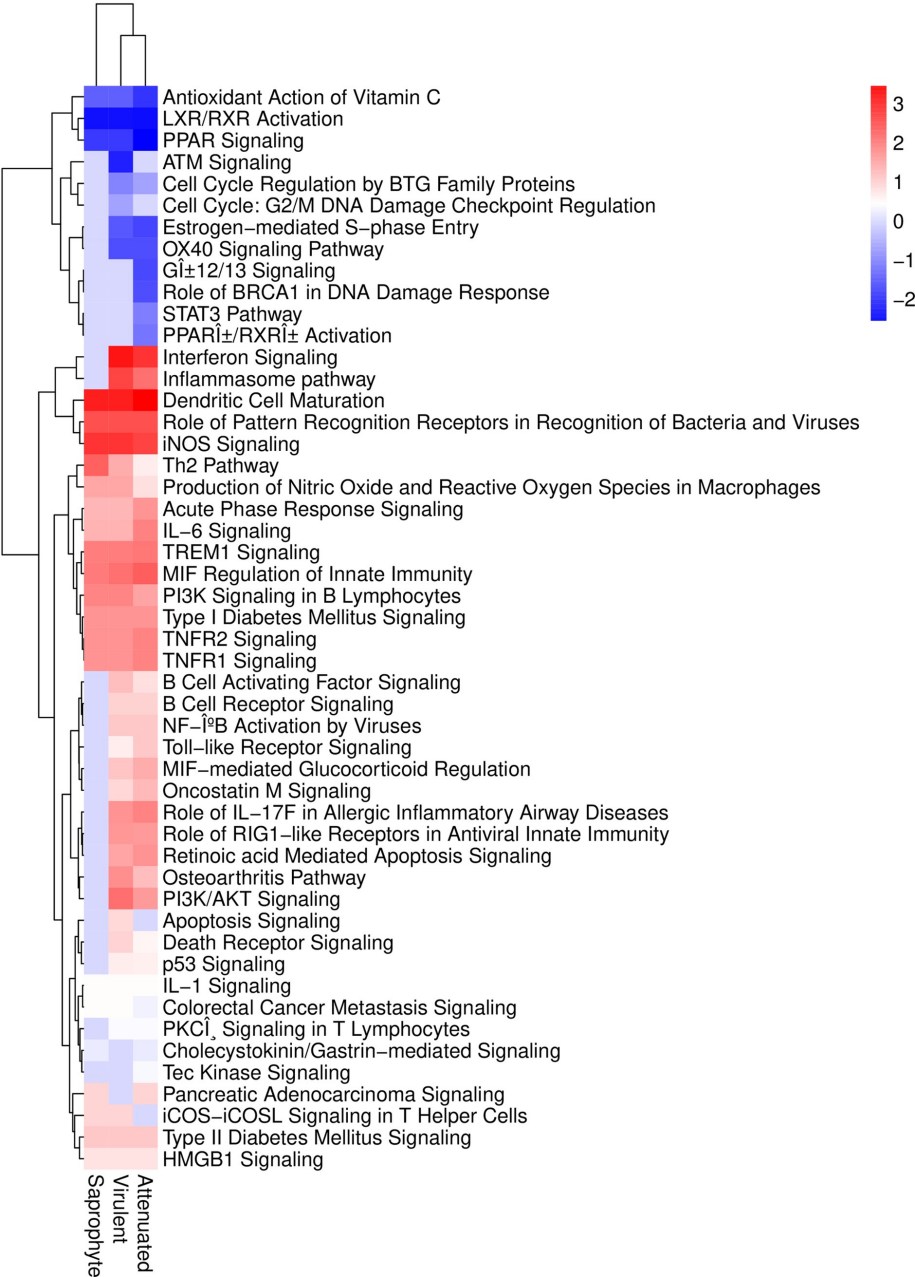
Heatmap of Canonical pathways predicted through z-score to be inhibited or activated in macrophages, at six hours of infection by different strains of Leptospira spp. Activated pathways are plotted in red color and inhibited pathways in blue. (n = 3/treatment; p-value < 0.01).

Other canonical pathways related to the innate immune system were a common response to all strains (Fig 7), the acute phase response signaling pathway, iNOS, IL-6, IL-1, TNFR1/TNFR2, MIF-regulation of innate immunity and HMGB-1 signaling. Further, all three strains are proposed to negatively regulate the antioxidant action of vitamin C pathway, suggesting that *Leptospira* spp. infection could contribute to oxidative stress associated production of reactive oxygen species (ROS).

ROS-mediated intracellular oxidation is prevented by an antioxidant system, which includes low molecular weight antioxidants, such as vitamin C. This pathway is involved in cell process of survival, growth, proliferation and death [25].

In the culture-attenuated and virulent samples, Toll-like receptors, Interferon and inflammasome signaling pathways were significantly represented. Innate immune response is initiated by recognizing pathogens through pattern receptors as TLRs. Activation of these receptors is characterized by the massive production/release of proinflammatory mediators, such as cytokines, chemokines and interferons [26]. Our microarray results identified higher expression of TNF-α, IL-1β, IL-6 and iNOS in the virulent group. Similarly, Iskandar et al [24] verified that the presence of IL-6, IL-8 and IL-1β in serum from human patients with leptospirosisis associated with severity of disease. In fact, Schulte et al. [25] concluded that increased TNF-α, IL-1β and IL-6 can activate the coagulation system in endotoxemic models, suggesting that high concentrations of IL-6 is an indicator of septic shock and correlates with disease severity in leptospirosis [26].

In regards to signaling pathways regulated by mRNAs modulated specifically following infection with virulent *L*. *interrogans*, Apoptosis signaling was positively regulated by infection whereas ATM signaling and Cell Cycle: G2/M DNA Damage Checkpoint Regulation, responsible for cell cycle, DNA repair and apoptosis, were negatively modulated. Following DNA damage, cells must detect breaks and transiently block the cell cycle progression allowing time for repair [27]. Jin *et al* [28] concluded that the pathogenic *Leptospira* caused apoptosis between 3–6 hours after infection. Our data corrobarates their finding that virulent *Leptospira* could modulated apoptosis, with just 6 hours of infection, through inhibition of pathways responsible for DNA repair and cell cycle control, as well as by inhibition the BCL-2 (anti-apoptotic gene) in turn leading to DNA damage and degradation. A previous study from our group has shown that BCL2 is a potentially down-regulated by mmu-mir-7667-3p following infection with *L*. *interrogans*, suggesting that cell survival could be compromissed after macrophages infection by the spirochete [29].

Leptospiral infection in macrophages induces a dependent p53/p21 cell cycle arrest [30]. We verified that the p53 target pathway signaling is regulated after virulent infection by modulation of mRNAs in murine monocyte-machrophages. Homotetrameric transcription factor p53 regulates 500 target genes, thereby controlling a broad range of cellular processes including cell cycle arrest, cell senescence, DNA repair, metabolic adaptation and cell death [31].

Further results from our study support the idea of cellular apoptosis Caspase-3 and 8 were elevated in all three infected macrophages, regardless of the pathogenicity notwithstanding, a more pronounced upregulation was induced by the with virulent and attenuated inoculum of *Leptospira*. Whether macrophage apoptosis induction by *Leptospira* is form of evasion mechanism or a host defense response to infection, preventing the spread of infection Jin et al., [28] is still up for debate.

Cytokines represent a group of proteins that promote communication between cells, and their activation is through differentiation, receptor expression and cell-mediated immunity [32]. This suggests that the virulence factors, expressed or not during the process of infection of *in vitro* macrophages can guide cell response. In other words, virulent, culture-attenuated and non-pathogenic samples of *Leptospira* should be able to activate the murine macrophages, and the gene expression elicited as a result of infection, is dependent on strain virulence samples. Our results revealed a quantitative and qualitative association of gene expression with the virulence strains, with the virulent *L*. *interrogans* upregulating genes related to acute infection and cellular autophagy, unlike the culture-attenuated and saprophytic strains.

A comprehensive overview of gene expression patterns after infection by virulent, culture-attenuated and saprophytic *Leptospira* spp. strains revealed that inflammation and immune response, cytokine signaling, DNA repair, cell movement, death and cell survival were significantly activated following 6 hours of infection. Results demonstrated a group of genes is responsive to antigens present in the genus *Leptospira*, regardless of virulence, whereas species and virulence-specific gene expression was also elicited in the infected macrophages.

## Methods

### Ethics statement

The present study was approved by the Research Ethics Committee of São Paulo State University (FMVA- UNESP), under the protocol number 2015–00895. No animal experimentation was performed in the experiments described herein.

### Leptospiral strains

Samples of virulent strain *L*. *interrogans* sorovar Copenhageni (FIOCRUZ L1-130), attenuated strain *L*. *interrogans* sorovar Copenhageni M20 and saprophyte strain *L*.*biflexa* sorovar Patoc (FIOCRUZ—Patoc I) that we used in this study were donated by the Laboratory of Bacterial Zoonosis, Department of Preventive Veterinary Medicine and Animal Health of School of Veterinary Medicine and Animal Science, University of São Paulo (FMVZ/USP). All strains were incubated at 30°C in Fletcher semi-solid culture medium.

### Macrophage culture

Murine monocyte-macrophage cells (*Mus musculus* monocyte-macrophage cell line J774A.1), provided by the Paul Ehrlich cell bank (Rio de Janeiro, Brazil), was used as described [26]. Cells were maintained at 37° C, 5% CO2 in RPMI-1640 media (Sigma, USA) supplemented with 10% heat-inactivated fetal bovine serum (Gibco, USA), 100 ug/mL streptomycin (Sigma Chemical Co St.Louis, MO), 0.03% L-glutamine solution (Sigma) and 100 UI/mL of penicillin, in 6-well cell culture plates (3cm/well) until confluency [22,29].

### Infection of macrophages

After the formation of confluent monolayer cells, they were washed three times in sterile phosphate buffer solution (pH 7.2) for removal of antibiotics and non-adherent cells. Bacteria were harvested by centrifugation and resuspended in RPMI-1640 media (Sigma). Cells were then infected (100:1 bacteria:cell) with *L*. *interrogans* L1-130 (virulent strain), *L*. *interrogans* M20 (culture-attenuated strain), *L*. *biflexa* Patoc I (saprophyte strain), as previously described [33]. Non-infected groups and non-infected macrophages were used as controls. All infected cells, in biological triplicates, were carried in fresh RPMI-1640, devoid of antibiotics, for 6h at 37° C, 5% CO_2_. Rate of infection did not differ between strains. At the end of the 6-hour period of infection, RNA extraction was immediately performed.

### RNA extraction and quantification

Total RNA (n = 3/experimental group) was extracted from macrophages with RNeasy Mini Kit (Qiagen, USA) according to manufacturer’s instructions. RNA samples were immediately stored at -80°C. The quantification was performed using a NanoDrop (ND-2000 spectrophotometer, Thermo Scientific, Wilmington, DE, USA) and the samples quality was assessed using capillary electrophoresis (Bioanalyzer 2100 Agilent, Santa Clara, CA, USA). All samples used for microarray analysis had a RIN of 10 (Quality data is available on our data descriptor)[22].

### Transcriptome array and quality control

A WT PLUS Reagent Kit was used to prepare the RNA samples for whole transcriptome expression analysis with Mouse Genome 2.1 ST Arrays Strip Affymetrix (Santa Clara, CA, USA), according to the manufacturer's protocols. Briefly, 100 ng of control RNA sample (Hela cells) was prepared to contain spiked in Poly-A RNA controls (lys, phe, thr and dap) absent in eukaryotic cells and mixed together with RNA samples to generate cDNA. After the amplification process, final cDNA was purified, quantified, fragmented and then labeled for hybridization to the strips, for 20h at 48°C in the hybridization oven. Finally, strips were processed using the GeneAtlas Hybridization, Wash, Stain Kit for WT Array Strips (Affymetrix) and scanned using the GeneAtlas® System (Affymetrix) generating the raw cell files. Raw intensity values in the cell files were background corrected, log2 transformed and then quantile normalized by the software Expression Console (Affymetrix) using the Robust Multi-array Average (RMA) algorithm.

### Identification of differentially expressed genes and functional enrichment

In order to identify differentially expressed genes, we utilized the software Transcriptome Analysis Console (Affymetrix), where statistical analysis was performed by one-way ANOVA (fold change ± 2, FDR corrected p<0.05). For the purpose of functional enrichment of the expression profiles obtained for each treatment, we used the Ingenuity Pathway Analysis (IPA) software (Qiagen).

### Validation of transcriptome results by qRT-PCR

For the validation of gene expression of selected genes in infected macrophages (saprophyte, culture-attenuated and virulent strains) and non-infected control macrophages, RNA samples were reverse transcribed (1μg of total RNA/sample) using the Moloney Murine Leukemia Virus (MML-V) enzyme (Life Technologies) and Oligo-dT Primers. All primers were designed to span at least one intron, to avoid repeat regions and similarities to other non-specific genomic regions. Mouse genome sequence, available on the University of California, Santa Cruz (UCSC) Genome Browser, was employed for primer design, using the Primer3 program [34]. PCR was performed using a Stratagene QPCR Systems Mx3005P (Agilent Technologies, Santa Clara, CA, USA) using the QuantiTect SYBR Green PCR kit (Qiagen). Expression levels were determined using standard curves for all genes at each individual run, and the expression of the candidate gene is presented as a ratio to an unregulated endogenous control (*β*-actin).

### Statistical analysis

Differential expression of each gene was determined by one-way ANOVA with two criteria, a fold change of ±2 comparing all infected groups to the non-infected control and a Benjamini-Hochberg (BH) corrected p-value (FDR)<0.05. For pathways enrichment analysis on the Ingenuity Pathway Analysis (IPA) software (Qiagen), multiple testing was also (BH corrected (p<0.05). Real-time PCR data were analyzed using least-squares analysis of variance and the general linear model procedures of SAS (SAS Institute, Cary, NC, USA; p <0.01). Comparison of means was done using Duncan's multiple range test.

## Supporting information

S1 Table DEGs (gene symbol) modulated by macrophages at 6h of infection by different strains of *Leptospira* spp. (DOC)

S1 Fig Top Canonical Pathways modulated by macrophages at 6h of infection by different strains of *Leptospira* spp. Canonical pathway expression in strains saprophyte, culture-attenuated and virulent compared with control. Using Ingenuity Pathway Analysis (IPA), the treatments groups were compared with control groups on differentially expressed genes with z-score that evaluate activation (positive score-orange) or down-regulation (negative score-blue). The bars reflect the p value for each pathway. The p value measures the likelihood that association between the differentially expressed genes in the dataset and the pathway is due to random. The smaller the p value, the taller the bar in the figure, and the less likely the association is due to random chance. All the pathways represented had p values > 1.3 (equivalent to a p-value <0.01) calculated by the Benjamini–Hochberg method and were considered statistically significant. **Panel A**) Pathways that were predicted to be activated or inhibited in saprophyte when compared to control groups. **Panel B)** Pathways that were predicted to be activated or inhibited in culture-attenuated when compared to control groups. **Panel C)** Pathways that were predicted to be activated or inhibited in virulent when compared to control groups.
(TIF)
