# Supplementary material for: Gene expression is associated with virulence in murine macrophages infected with Leptospira spp
Source: PLoS One. 2019 Dec 4;14(12):e0225272. doi: 10.1371/journal.pone.0225272 (PMC6892507; doi:10.1371/journal.pone.0225272)
Supplement: S1 Table — (DOC) [file pone.0225272.s001.doc]

| Vir vs. Ct | Att vs. Ct | Sap vs. Ct | Vir vs. Att | Vir vs. Sap | Vir vs. Att vs. Sap | Att vs. Sap |
| --- | --- | --- | --- | --- | --- | --- |
| Rpa1 | 4930522L14Rik | Abcg1 | Ly6a | Arrdc3 | Ccl17 | Emp1 |
| Xrcc3 | Heatr9 | Rab32 | Dtl |  | Rnf144b | Arl11 |
| Helb | Snapc5 | Nadk | Gbp9 |  | Mocs1 | Mr1 |
| Bambi-ps1 | Gpr85 | Cln5 | Fcgr1 |  | Gm21188 | Hmox1 |
| Psmb9 | Cenpk | Psd3 | Ifi47 |  | Pstpip2 | Tma16 |
| C130026I21Rik | Ppm1k | Cd80 | Olfr56 |  | Gbp2 | Pim3 |
| Gm35498 | Haus4 | Lpcat2 | Map3k8 |  | Nlrp3 | Gm33049 |
| Haus1 | Pxmp4 | 9930111J21Rik2 | B430306N03Rik |  | Hcar2 | Gpr18 |
| Mitf | Dpy19l4 | Rrs1 | Arrb1 |  | Il1a |  |
| Sav1 | Kdelc2 | Slc46a3 | Parp12 |  | Gm26714 |  |
| Snx25 | Aaed1 | Ptpro | Usp25 |  | Slc7a2 |  |
| Slc25a37 | Loxl3 | Clec4n | Gm12250 |  | Epsti1 |  |
| Tgfb3 | Mgea5 | Eps8 | Pxk |  | Bcl2a1b |  |
| Zcchc6 | Zfc3h1 | Zswim7 | Asap1 |  | Bcl2a1a |  |
| Rap2c | Parp8 | Mastl | Tnfsf10 |  | Ccl12 |  |
| Plekhg3 | Gm5154 | Arl5c | Exo1 |  | Nav1 |  |
| Ints7 | Hist1h2bk | Serinc2 | Mcm3 |  | Tlr2 |  |
| Pola2 | Syk | Denr | Trim30c |  | Slpi |  |
| Cnn3 | Tmem8 | Sowahc | Mxd1 |  | Gmnn |  |
| Fam111a | Dut | Ptger4 | Nmi |  | Tpcn1 |  |
| Bend4 | Hist1h2bh | Gm10134 | Appl2 |  | Nfkbia |  |
| Themis2 | Sertad1 | Ptchd1 | Mcm5 |  | P3h2 |  |
| Zmynd15 | Zak |  | Aida |  | Arhgef3 |  |
| E2f2 | Id1 |  | Mov10 |  | Osm |  |
| Adrb2 | Mis18a |  | Nsl1 |  | Skil |  |
| Raph1 | Fat3 |  | Rps6ka5 |  | Mcm4 |  |
| Whamm | Dock1 |  | Diaph2 |  | Esco2 |  |
| Pgp | Tipin |  | Parp10 |  | Smpdl3b |  |
| Eif2ak3 | Camkk2 |  | Plec |  | Saa3 |  |
| Narf | Kcnj2 |  | Gm17757 |  | Gsap |  |
| Azi2 | Ctns |  | Gm4070 |  | Nfkbiz |  |
| Fut11 | Etv5 |  | Tap1 |  | Zdhhc14 |  |
| Ska3 | Crip1 |  | 4930579G24Rik |  | Ulbp1 |  |
| Parp11 | Tnfrsf23 |  | Ezr |  | Gbp5 |  |
| Ccne1 | Wbscr27 |  | Lgals9 |  | Gas6 |  |
| Pdpr | 1700025G04Rik |  | Xpr1 |  | Irak3 |  |
| Dck | Ms4a14 |  | Slfn1 |  | Il6 |  |
| Chtf18 | Hist1h4h |  | Capn2 |  | St8sia4 |  |
| Mxd4 | Cenpf |  | Mndal |  | Phka2 |  |
| Aff1 | Wdr62 |  | Usb1 |  | Cxcl2 |  |
| Abi2 | Arap2 |  | Phf11b |  | Chaf1b |  |
| Sepw1 | Aoah |  | 5031439G07Rik |  | Ifi205 |  |
| Lcp2 | Dhcr7 |  | LOC102636558 |  | Bst2 |  |
| Pip5k1a | Cuta |  | Enpp4 |  | Arhgap18 |  |
| Kif21b | Pgap1 |  | Hacl1 |  | Ehd1 |  |
| Tor1aip1 | Samd9l |  | Cacna1a |  | Gpr84 |  |
| Ndufaf7 | E2f5 |  | Nfkbie |  | Msh6 |  |
| Rhod | Rgs1 |  | Smarca2 |  | Gbp7 |  |
| Dag1 | Rab3il1 |  | Timp2 |  | Hdac5 |  |
| LOC102638047 | Zfp36l1 |  | Slfn4 |  | Wdhd1 |  |
| LOC100041877 | Mthfd2 |  | Uba7 |  | Cxcl11 |  |
| Gpr162 |  |  | Sp100 |  | Fam13a |  |
| Kdm7a |  |  | Bhlhe40 |  | Plxnc1 |  |
| Man2a2 |  |  | Nfic |  | Lcn2 |  |
| Nfatc1 |  |  | Gbp4 |  | Camk1 |  |
| Fbxw17 |  |  | Znfx1 |  | Irg1 |  |
| Rbm4b |  |  | Frmd4a |  | C3 |  |
| Fhod1 |  |  | Prkch |  | Tnf |  |
| Marveld1 |  |  | Wdr91 |  | Gm5424 |  |
| Idh1 |  |  | Mmp13 |  | Ass1 |  |
| Proser3 |  |  | Phf11a |  | Traf1 |  |
| 0610009O20Rik |  |  | Cdca7l |  | Nav2 |  |
| Scarb1 |  |  | Samhd1 |  | Crlf3 |  |
| Rfc4 |  |  | Rnf213 |  | Nos2 |  |
| Tgfbr2 |  |  | Cybb |  | Il1rn |  |
| Smtn |  |  | Rffl |  | Marco |  |
| Crot |  |  | Lgals3bp |  | Il1rl1 |  |
| Stard4 |  |  | Slc16a7 |  | Ccl7 |  |
| Ccnj |  |  | Ube2l6 |  | Wdr76 |  |
| Tonsl |  |  | Helz2 |  | Ifi44l |  |
| Fcgr4 |  |  | Cxcr3 |  | Tlr5 |  |
| Slc7a5 |  |  | Msh2 |  | Irf7 |  |
| Sorbs1 |  |  | Trim30d |  | Ndrg1 |  |
| Ddit3 |  |  | Nod2 |  | Tecpr1 |  |
| Gm16340 |  |  | Igtp |  | Ccl5 |  |
| Il27 |  |  | Gm12764 |  | Zhx2 |  |
| Serpina3f |  |  | Il15 |  | Car2 |  |
| Serpina3g |  |  | Gm38510 |  | Slc31a2 |  |
| Fam134b |  |  | Csprs |  | D430020J02Rik |  |
| Ppp4r2 |  |  | Mtss1 |  | Tnfrsf1b |  |
| Sat1 |  |  | Abcc5 |  | Id3 |  |
| Tm7sf3 |  |  | Mms22l |  | Gm12840 |  |
| Gatsl2 |  |  | Rrm2 |  | Socs3 |  |
| Mgl2 |  |  | N4bp1 |  | Gbp3 |  |
| Pcsk2 |  |  | Gins2 |  | Csf3 |  |
| Rasgef1b |  |  | Gm18853 |  | Pilra |  |
| Slc35g1 |  |  | Tlr3 |  | Ifi202b |  |
| St3gal1 |  |  | Psmb10 |  | Oasl1 |  |
| Ttk |  |  | Epb41 |  | Dhfr |  |
| Pxdc1 |  |  | Stat1 |  | Cd69 |  |
| Fam46c |  |  | Tubgcp2 |  | Icosl |  |
| Gm38530 |  |  | Cdk19 |  | Ier3 |  |
| Slfn3 |  |  | Apol9a |  | Dscc1 |  |
| Spc25 |  |  | Oas2 |  | Zbp1 |  |
| Wdr43 |  |  | Iigp1 |  | Cd40 |  |
| Neurl1b |  |  | Socs1 |  | Mfsd6 |  |
| Sla |  |  | Ttc7b |  | Hck |  |
| Vsir |  |  | Galnt12 |  | Ptgs2 |  |
| Mblac2 |  |  | Stim2 |  | Slc37a2 |  |
| Mdm2 |  |  | Zgrf1 |  | Topbp1 |  |
| Trappc1 |  |  | Rgs18 |  | Mfsd11 |  |
| Zc3hav1l |  |  | Pole2 |  | Ikbke |  |
| Slc12a9 |  |  | Nfkb1 |  | Rhbdf2 |  |
| Parp1 |  |  | Setdb2 |  | Ccrl2 |  |
|  |  |  | Phf11c |  | Tagap |  |
|  |  |  | 4933412E12Rik |  | Vasp |  |
|  |  |  | Uhrf1 |  | Cpd |  |
|  |  |  | Tmem18 |  | Dnmt1 |  |
|  |  |  | Ifi35 |  | Sod2 |  |
|  |  |  | Tspan17 |  | Nrcam |  |
|  |  |  | Cxcl10 |  | Hal |  |
|  |  |  | Gphn |  | Cdc6 |  |
|  |  |  | Mcm8 |  | Lsr |  |
|  |  |  | Pyhin1 |  | Ccl2 |  |
|  |  |  | Oas1g |  | Ncapg2 |  |
|  |  |  | Neo1 |  | Tnip1 |  |
|  |  |  | Casp4 |  | Ang |  |
|  |  |  | Plxdc1 |  | Rnase4 |  |
|  |  |  | Mcm2 |  | Jag1 |  |
|  |  |  | Pkmyt1 |  | Vav3 |  |
|  |  |  | A530040E14Rik |  | Bcl3 |  |
|  |  |  | Prim2 |  | Clec4e |  |
|  |  |  | Nfxl1 |  | Tfdp2 |  |
|  |  |  | Acox3 |  | Ccne2 |  |
|  |  |  | Jdp2 |  | Ankrd37 |  |
|  |  |  | Gvin1 |  | Tmem71 |  |
|  |  |  | Nbeal2 |  | Mcm6 |  |
|  |  |  | Nrp2 |  | Oasl2 |  |
|  |  |  | Gbp10 |  | Daglb |  |
|  |  |  | Ogfr |  | Ctc1 |  |
|  |  |  | Peli1 |  | Rhoq |  |
|  |  |  | Gm40824 |  | Gm41656 |  |
|  |  |  | Cadps2 |  | Glrx |  |
|  |  |  | Insig1 |  | Zc3h12a |  |
|  |  |  | Wee1 |  | Bcl2a1d |  |
|  |  |  | Adora2b |  | Car13 |  |
|  |  |  | Stat2 |  | Pim1 |  |
|  |  |  | Atad2 |  | Cers6 |  |
|  |  |  | Kif13a |  | Snx10 |  |
|  |  |  | Zfp36 |  | Lig1 |  |
|  |  |  | Pnpt1 |  | Junb |  |
|  |  |  | H2-T22 |  | Adgre1 |  |
|  |  |  | H2-T9 |  | Klhl24 |  |
|  |  |  | Eepd1 |  | Hmga2-ps1 |  |
|  |  |  | Ifi203 |  | Cdc42ep2 |  |
|  |  |  | Rab27a |  | Stk40 |  |
|  |  |  | Chek2 |  | Tm6sf1 |  |
|  |  |  | Parp14 |  | Ighm |  |
|  |  |  | Dsn1 |  | Gm17035 |  |
|  |  |  | Gm7609 |  | Antxr1 |  |
|  |  |  | Bcl6 |  | Il18rap |  |
|  |  |  | Ccl22 |  | Mkl1 |  |
|  |  |  | Gm8989 |  | Pip4k2a |  |
|  |  |  | Zfand4 |  | Gbp2b |  |
|  |  |  | Dtx3l |  | Gnpda1 |  |
|  |  |  | Glipr2 |  | Tbc1d8 |  |
|  |  |  | Isg15 |  | Arhgap6 |  |
|  |  |  | Ifit3b |  | Csf1 |  |
|  |  |  | Gm8979 |  | Icam1 |  |
|  |  |  | Ddx58 |  | Trex1 |  |
|  |  |  | Csrnp1 |  | Atrip |  |
|  |  |  | Irgm2 |  | Fanci |  |
|  |  |  | Dhx58 |  | Jak2 |  |
|  |  |  | Mlkl |  | Ctdspl |  |
|  |  |  | Bst1 |  | Hyal1 |  |
|  |  |  | Slc4a7 |  | Nat6 |  |
|  |  |  | Zfp36l2 |  | B4galt6 |  |
|  |  |  | Cd274 |  | Rab20 |  |
|  |  |  | Slamf8 |  | Gch1 |  |
|  |  |  | Tnfsf15 |  | Procr |  |
|  |  |  | Nampt |  | Nfkbid |  |
|  |  |  | Daxx |  | Slc39a10 |  |
|  |  |  | Gcnt2 |  | Pole |  |
|  |  |  | Apol9b |  | Syne1 |  |
|  |  |  | Cd84 |  | Rapgef2 |  |
|  |  |  | Sp140 |  | Fas |  |
|  |  |  | Ifih1 |  | Ifi204 |  |
|  |  |  | Gm8995 |  | Ank |  |
|  |  |  | Chaf1a |  | Ralgds |  |
|  |  |  | Ascc3 |  | Ms4a6c |  |
|  |  |  | Slfn5 |  | St18 |  |
|  |  |  | Gins1 |  | Mcm7 |  |
|  |  |  | H2-T23 |  | Paqr4 |  |
|  |  |  | Aftph |  | Il1b |  |
|  |  |  | Ifit1bl1 |  | Tet2 |  |
|  |  |  | Mkx |  | Rnasel |  |
|  |  |  | Rnf150 |  | Antxr2 |  |
|  |  |  | H2afv |  | Tcf19 |  |
|  |  |  | Tmem218 |  | Adhfe1 |  |
|  |  |  | Pml |  | Dram1 |  |
|  |  |  | Cdkn3 |  | Gm40733 |  |
|  |  |  | Gm16094 |  | Brca1 |  |
|  |  |  | Trafd1 |  | LOC102634900 |  |
|  |  |  | Ttyh3 |  | Gm16685 |  |
|  |  |  | Mmgt1 |  | Cxcr4 |  |
|  |  |  | Naa25 |  | Pou2f2 |  |
|  |  |  | LOC100503923 |  | Rasgrp3 |  |
|  |  |  | Dnajc9 |  | Zfp367 |  |
|  |  |  | Polh |  | Tnfaip2 |  |
|  |  |  | Zfpm1 |  | Gm7334 |  |
|  |  |  | Art3 |  | Btg3 |  |
|  |  |  | Ptgs1 |  | Kitl |  |
|  |  |  | Herc6 |  | Txnrd1 |  |
|  |  |  | Bcl2 |  | Hist1h1b |  |
|  |  |  | Il1f6 |  | Rad54l |  |
|  |  |  | Map1lc3b |  | Siglece |  |
|  |  |  | Utp14b |  | Rab11fip1 |  |
|  |  |  | Acsl3 |  | Rnf19b |  |
|  |  |  | Arid5b |  | Serpine1 |  |
|  |  |  | Fbxo5 |  | Rasa3 |  |
|  |  |  | AW112010 |  | Sgsh |  |
|  |  |  | Cenph |  | Oaf |  |
|  |  |  | Slbp |  | Rtn2 |  |
|  |  |  | Maml3 |  | Clec4d |  |
|  |  |  | Plekhn1 |  | Lpin1 |  |
|  |  |  | Fndc3a |  | Itga6 |  |
|  |  |  | Arel1 |  | Zc3h12c |  |
|  |  |  | Cdk2 |  | Rb1 |  |
|  |  |  | Mknk2 |  | Brip1 |  |
|  |  |  | Trim30a |  | Lacc1 |  |
|  |  |  | A530064D06Rik |  | Myo1f |  |
|  |  |  | Cd28 |  | Cenpn |  |
|  |  |  | Tmem171 |  | Mrpl52 |  |
|  |  |  | Il13ra1 |  | Tmem154 |  |
|  |  |  | Fads1 |  | Il1f9 |  |
|  |  |  | Stx11 |  | Il4ra |  |
|  |  |  | St3gal3 |  | Srgap3 |  |
|  |  |  | Tapbp |  | Igsf6 |  |
|  |  |  | Gadd45g |  | Deptor |  |
|  |  |  | Rrm1 |  | Rdh10 |  |
|  |  |  | Atrn |  | Pilrb1 |  |
|  |  |  | Nlrc5 |  | Ctsh |  |
|  |  |  | Hip1 |  | Plaur |  |
|  |  |  | Mx1 |  | Psmd10 |  |
|  |  |  | Mcoln2 |  | Cerk |  |
|  |  |  | Tmod1 |  | Fam63b |  |
|  |  |  | Ints10 |  | Neil3 |  |
|  |  |  | Hdc |  | Nrm |  |
|  |  |  | Sesn2 |  | Slc7a11 |  |
|  |  |  | Mnda |  | Rgs2 |  |
|  |  |  | Tmem19 |  | Hk2 |  |
|  |  |  | Hist4h4 |  | Tec |  |
|  |  |  | Impa2 |  | Map2k6 |  |
|  |  |  | H2-T24 |  | Lbh |  |
|  |  |  | Lilra5 |  | Cebpb |  |
|  |  |  | Irak2 |  | Gm23428 |  |
|  |  |  | Siglec1 |  | Cytip |  |
|  |  |  | Alms1 |  | Ube2e2 |  |
|  |  |  | Trim26 |  | Tnfaip3 |  |
|  |  |  | Dgkh |  | Ptprj |  |
|  |  |  | Rad51ap1 |  | Gm38718 |  |
|  |  |  | Gna12 |  | Gm39869 |  |
|  |  |  | Parp9 |  | Abcc1 |  |
|  |  |  | Ticrr |  | Ccl3 |  |
|  |  |  | Adar |  | Ptpn22 |  |
|  |  |  | LOC101055758 |  | Plau |  |
|  |  |  | Gm7592 |  | Rbl2 |  |
|  |  |  | Haus5 |  | Gpr183 |  |
|  |  |  | Atad5 |  | P2ry13 |  |
|  |  |  | Xaf1 |  | 1810011O10Rik |  |
|  |  |  | Lpar5 |  | Dgat2 |  |
|  |  |  | Phtf1 |  | Plk3 |  |
|  |  |  | Clspn |  | Trem1 |  |
|  |  |  | Tmem67 |  | St6gal1 |  |
|  |  |  | Cp |  | Snx24 |  |
|  |  |  | Zeb1 |  | Tlr13 |  |
|  |  |  | C130050O18Rik |  | Mef2c |  |
|  |  |  | Tmem184c |  | Cd300lb |  |
|  |  |  | Abtb2 |  | Kctd12 |  |
|  |  |  | Larp1 |  | Txnip |  |
|  |  |  | Zcchc2 |  | Cd302 |  |
|  |  |  | Ifi44 |  | Efr3b |  |
|  |  |  | Il23r |  | Xylt1 |  |
|  |  |  | Hist1h2ag |  | Brca2 |  |
|  |  |  | Dfna5 |  |  |  |
|  |  |  | Ankrd44 |  |  |  |
|  |  |  | Tlk2 |  |  |  |
|  |  |  | Fam105a |  |  |  |
|  |  |  | Il18 |  |  |  |
|  |  |  | LOC105246895 |  |  |  |
|  |  |  | Gm11772 |  |  |  |
|  |  |  | Rfc2 |  |  |  |
|  |  |  | Papd7 |  |  |  |
|  |  |  | St3gal6 |  |  |  |
|  |  |  | Nuf2 |  |  |  |
|  |  |  | Tdrd7 |  |  |  |
|  |  |  | Pde10a |  |  |  |
|  |  |  | Ncapd3 |  |  |  |
|  |  |  | Tmem254b |  |  |  |
|  |  |  | Tmem254c |  |  |  |
|  |  |  | Tmem254a |  |  |  |
|  |  |  | Oas1b |  |  |  |
|  |  |  | Gpr155 |  |  |  |
|  |  |  | Xylt2 |  |  |  |
|  |  |  | S1pr1 |  |  |  |
|  |  |  | Pcdh7 |  |  |  |
|  |  |  | Stk17b |  |  |  |
|  |  |  | Rmnd5a |  |  |  |
|  |  |  | Itga5 |  |  |  |
|  |  |  | Cmpk2 |  |  |  |
|  |  |  | Mx2 |  |  |  |
|  |  |  | Inhba |  |  |  |
|  |  |  | Malt1 |  |  |  |
|  |  |  | Kif2c |  |  |  |
|  |  |  | Prkag2 |  |  |  |
|  |  |  | Havcr2 |  |  |  |
|  |  |  | LOC100041903 |  |  |  |
|  |  |  | Gm15433 |  |  |  |
|  |  |  | Cdkn1a |  |  |  |
|  |  |  | Cflar |  |  |  |
|  |  |  | Kif14 |  |  |  |
|  |  |  | Alg10b |  |  |  |
|  |  |  | Gbp11 |  |  |  |
|  |  |  | Ddx60 |  |  |  |
|  |  |  | Gm4951 |  |  |  |
|  |  |  | Pdxk |  |  |  |
|  |  |  | Kcnk13 |  |  |  |
|  |  |  | Abca1 |  |  |  |
|  |  |  | Atp13a3 |  |  |  |
|  |  |  | Mybl2 |  |  |  |
|  |  |  | Abcd1 |  |  |  |
|  |  |  | Fgf11 |  |  |  |
|  |  |  | Ifit1 |  |  |  |
|  |  |  | Lmnb1 |  |  |  |
|  |  |  | Rcbtb2 |  |  |  |
|  |  |  | Ell2 |  |  |  |
|  |  |  | Isg20 |  |  |  |
|  |  |  | Tk1 |  |  |  |
|  |  |  | Chek1 |  |  |  |
|  |  |  | Galc |  |  |  |
|  |  |  | Lonrf3 |  |  |  |
|  |  |  | LOC101055663 |  |  |  |
|  |  |  | Mitd1 |  |  |  |
|  |  |  | Trim21 |  |  |  |
|  |  |  | Lgals8 |  |  |  |
|  |  |  | Adssl1 |  |  |  |
|  |  |  | Itgal |  |  |  |
|  |  |  | Ms4a6b |  |  |  |
|  |  |  | Slamf7 |  |  |  |
|  |  |  | Irf1 |  |  |  |
|  |  |  | Hpgds |  |  |  |
|  |  |  | Tmem144 |  |  |  |
|  |  |  | Gtpbp2 |  |  |  |
|  |  |  | I830077J02Rik |  |  |  |
|  |  |  | Cdkn2c |  |  |  |
|  |  |  | Lrrc25 |  |  |  |
|  |  |  | 8430408G22Rik |  |  |  |
|  |  |  | Cmc2 |  |  |  |
|  |  |  | Nfat5 |  |  |  |
|  |  |  | Gm5431 |  |  |  |
|  |  |  | Tor3a |  |  |  |
|  |  |  | Slc25a22 |  |  |  |
|  |  |  | BC147527 |  |  |  |
|  |  |  | Cdca5 |  |  |  |
|  |  |  | Asf1b |  |  |  |
|  |  |  | Stil |  |  |  |
|  |  |  | Tmem64 |  |  |  |
|  |  |  | Toporsos |  |  |  |
|  |  |  | 1810011H11Rik |  |  |  |
|  |  |  | E2f1 |  |  |  |
|  |  |  | Klc4 |  |  |  |
|  |  |  | Cenpm |  |  |  |
|  |  |  | Nupr1 |  |  |  |
|  |  |  | Cdc45 |  |  |  |
|  |  |  | Epas1 |  |  |  |
|  |  |  | Pola1 |  |  |  |
|  |  |  | Add3 |  |  |  |
|  |  |  | Zufsp |  |  |  |
|  |  |  | E2f8 |  |  |  |
|  |  |  | Gm2427 |  |  |  |
|  |  |  | Ms4a6d |  |  |  |
|  |  |  | Ptafr |  |  |  |
|  |  |  | Ripk2 |  |  |  |
|  |  |  | Donson |  |  |  |
|  |  |  | Atp5o |  |  |  |
|  |  |  | Ttc39aos1 |  |  |  |
|  |  |  | Fancd2 |  |  |  |
|  |  |  | Stab1 |  |  |  |
|  |  |  | Slfn9 |  |  |  |
|  |  |  | Gm5454 |  |  |  |
|  |  |  | Mir155 |  |  |  |
|  |  |  | Adamts10 |  |  |  |
|  |  |  | Batf2 |  |  |  |
|  |  |  | Gm2666 |  |  |  |
|  |  |  | LOC100041708 |  |  |  |
|  |  |  | Ccna2 |  |  |  |
|  |  |  | Depdc1b |  |  |  |
|  |  |  | Ulk2 |  |  |  |
|  |  |  | Sass6 |  |  |  |
|  |  |  | Hadh |  |  |  |
|  |  |  | Cit |  |  |  |
|  |  |  | Kif15 |  |  |  |
|  |  |  | Prim1 |  |  |  |
|  |  |  | Man1c1 |  |  |  |
|  |  |  | Dhrs9 |  |  |  |
|  |  |  | Nt5dc2 |  |  |  |
|  |  |  | Ifit2 |  |  |  |
|  |  |  | Phf11d |  |  |  |
|  |  |  | Atf3 |  |  |  |
|  |  |  | Dpep2 |  |  |  |
|  |  |  | BC023105 |  |  |  |
|  |  |  | Ndc80 |  |  |  |
|  |  |  | Mical1 |  |  |  |
|  |  |  | Cpeb1 |  |  |  |
|  |  |  | Chac1 |  |  |  |
|  |  |  | Mfap3l |  |  |  |
|  |  |  | Aspm |  |  |  |
|  |  |  | Ctdsp2 |  |  |  |
|  |  |  | Mir26a-2 |  |  |  |
|  |  |  | Mir546 |  |  |  |
|  |  |  | Cdca7 |  |  |  |
|  |  |  | Slc30a1 |  |  |  |
|  |  |  | Usp18 |  |  |  |
|  |  |  | Ccl4 |  |  |  |
|  |  |  | Slfn2 |  |  |  |
|  |  |  | Fancm |  |  |  |
|  |  |  | Kif11 |  |  |  |
|  |  |  | Pdcd4 |  |  |  |
|  |  |  | Rassf2 |  |  |  |
|  |  |  | Ppa1 |  |  |  |
|  |  |  | Kremen1 |  |  |  |
|  |  |  | Mdc1 |  |  |  |
|  |  |  | Cfb |  |  |  |
|  |  |  | Ncaph |  |  |  |
|  |  |  | Kif23 |  |  |  |
|  |  |  | Bank1 |  |  |  |
|  |  |  | Gpsm2 |  |  |  |
|  |  |  | Chml |  |  |  |
|  |  |  | Slc17a5 |  |  |  |
|  |  |  | Ncapd2 |  |  |  |
|  |  |  | Mki67 |  |  |  |
|  |  |  | Rassf3 |  |  |  |
|  |  |  | Pcgf5 |  |  |  |
|  |  |  | Wfdc17 |  |  |  |
|  |  |  | Gpr137b |  |  |  |
|  |  |  | Fam46a |  |  |  |
|  |  |  | Hist2h2ab |  |  |  |
|  |  |  | 1190002N15Rik |  |  |  |
|  |  |  | Cth |  |  |  |
|  |  |  | Slc7a1 |  |  |  |
|  |  |  | Irgm1 |  |  |  |
|  |  |  | Hist1h2af |  |  |  |
|  |  |  | Dusp5 |  |  |  |
|  |  |  | Foxred2 |  |  |  |
|  |  |  | Il18bp |  |  |  |
|  |  |  | Glul |  |  |  |
|  |  |  | Mir8114 |  |  |  |
|  |  |  | Sesn1 |  |  |  |
|  |  |  | Acsl1 |  |  |  |
|  |  |  | Nt5c2 |  |  |  |
|  |  |  | Primpol |  |  |  |
|  |  |  | Pold1 |  |  |  |
|  |  |  | Foxm1 |  |  |  |
|  |  |  | Bard1 |  |  |  |
|  |  |  | Melk |  |  |  |
|  |  |  | Tmem260 |  |  |  |
|  |  |  | Gm21370 |  |  |  |
|  |  |  | Hist1h2ab |  |  |  |
|  |  |  | Tbc1d4 |  |  |  |
|  |  |  | Hmha1 |  |  |  |
|  |  |  | Fgd4 |  |  |  |
|  |  |  | Morc3 |  |  |  |
|  |  |  | Hist2h2be |  |  |  |
|  |  |  | March5 |  |  |  |
|  |  |  | Slc2a6 |  |  |  |
|  |  |  | Fmnl2 |  |  |  |
|  |  |  | Fignl1 |  |  |  |
|  |  |  | Lpar6 |  |  |  |
|  |  |  | Etnk1 |  |  |  |
|  |  |  | Lpl |  |  |  |
|  |  |  | Nfkbib |  |  |  |
|  |  |  | Sptssa |  |  |  |
|  |  |  | Mir1892 |  |  |  |
|  |  |  | Flnb |  |  |  |
|  |  |  | Cyp2r1 |  |  |  |
|  |  |  | 1700006J14Rik |  |  |  |
|  |  |  | AA467197 |  |  |  |
|  |  |  | Mir147 |  |  |  |
|  |  |  | Rnf135 |  |  |  |
|  |  |  | Rsad2 |  |  |  |
|  |  |  | Kif18a |  |  |  |
|  |  |  | Ska2 |  |  |  |
|  |  |  | Il15ra |  |  |  |
|  |  |  | Tap2 |  |  |  |
|  |  |  | Cptp |  |  |  |
|  |  |  | Lyl1 |  |  |  |
|  |  |  | Arid5a |  |  |  |
|  |  |  | Tmem65 |  |  |  |
|  |  |  | Gm6904 |  |  |  |
|  |  |  | Adap2 |  |  |  |
|  |  |  | Kntc1 |  |  |  |
|  |  |  | Scimp |  |  |  |
|  |  |  | Dcstamp |  |  |  |
|  |  |  | Kif18b |  |  |  |
|  |  |  | Dtymk |  |  |  |
|  |  |  | 2810417H13Rik |  |  |  |
|  |  |  | F830016B08Rik |  |  |  |
|  |  |  | Arntl |  |  |  |
|  |  |  | Wwp1 |  |  |  |
|  |  |  | Slc9a9 |  |  |  |
|  |  |  | Tjp2 |  |  |  |
|  |  |  | Clec7a |  |  |  |
|  |  |  | Mmp25 |  |  |  |
|  |  |  | Cbx5 |  |  |  |
|  |  |  | Trib3 |  |  |  |
|  |  |  | Birc5 |  |  |  |
|  |  |  | Gclm |  |  |  |
|  |  |  | 1500012F01Rik |  |  |  |
|  |  |  | Snord88c |  |  |  |
|  |  |  | Bcat1 |  |  |  |
|  |  |  | Casp1 |  |  |  |
|  |  |  | Snx29 |  |  |  |
|  |  |  |  |  |  |  |
|  |  |  |  |  |  |  |

**Supporting information**

**S1 Table. DEGs (gene symbol) modulated by macrophages at 6h of infection by different strains of *Leptospira* spp.**
