# Supplementary figures and images for: Gene expression is associated with virulence in murine macrophages infected with Leptospira spp
Source: PLoS One. 2019 Dec 4;14(12):e0225272. doi: 10.1371/journal.pone.0225272 (PMC6892507; doi:10.1371/journal.pone.0225272)

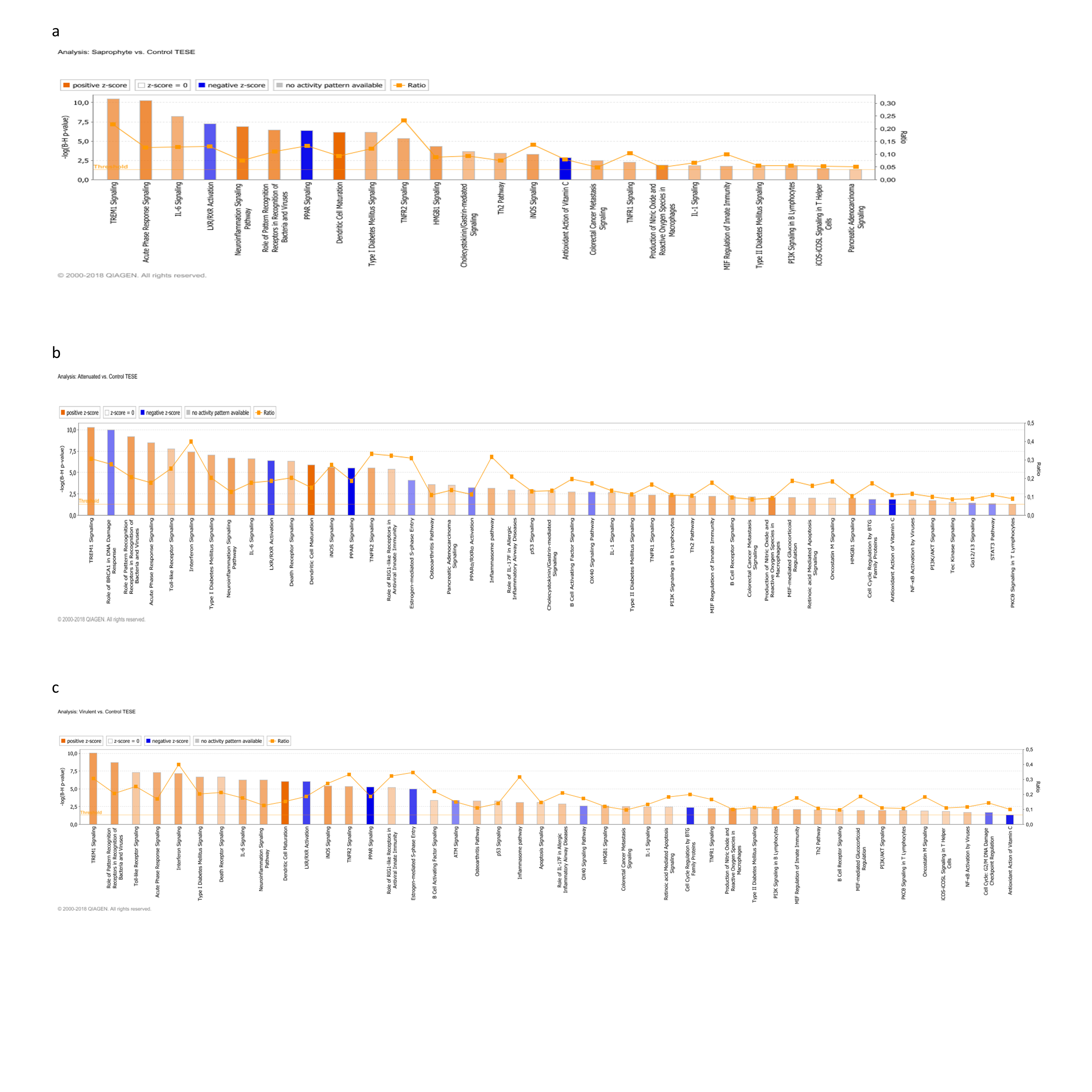

Supplement: S1 Fig — Canonical pathway expression in strains saprophyte, culture-attenuated and virulent compared with control. Using Ingenuity Pathway Analysis (IPA), the treatments groups were compared with control groups on differentially expressed genes with z-score that evaluate activation (positive score-orange) or down-regulation (negative score-blue). The bars reflect the p value for each pathway. The p value measures the likelihood that association between the differentially expressed genes in the dataset and the pathway is due to random. The smaller the p value, the taller the bar in the figure, and the less likely the association is due to random chance. All the pathways represented had p values > 1.3 (equivalent to a p-value <0.01) calculated by the Benjamini–Hochberg method and were considered statistically significant. Panel A) Pathways that were predicted to be activated or inhibited in saprophyte when compared to control groups. Panel B) Pathways that were predicted to be activated or inhibited in culture-attenuated when compared to control groups. Panel C) Pathways that were predicted to be activated or inhibited in virulent when compared to control groups. (TIF) [file pone.0225272.s002.tif]
